# Physiological and Proteomic Insights into Melatonin-Mediated Regulation of Copper Toxicity in the Crayfish *Procambarus clarkii*

**DOI:** 10.3390/ijms27125236

**Published:** 2026-06-09

**Authors:** Zaihang Yu, Xinyu Li, Le Zhang, He Lv, Yang Shen, Zhoufo Lu, Fangming Xu, Yi Chen, Xueting Zhong, Zhanqi Wang

**Affiliations:** 1 Zhejiang Provincial Key Laboratory of Biology of Crop Pathogens and Insects, College of Life Sciences, Huzhou Normal University, Huzhou 313000, China; yuzaihang6@163.com (Z.Y.); xinyu01230@163.com (X.L.); zhangle011122@163.com (L.Z.); sy18157382505@163.com (Y.S.); zhoufo_lu999@163.com (Z.L.); 19857223353@163.com (F.X.); 15280674928@163.com (Y.C.); zxt@zjhu.edu.cn (X.Z.); 2 Zhejiang Provincial Key Laboratory of Aquatic Resources Conservation and Development, College of Life Sciences, Huzhou Normal University, Huzhou 313000, China; 02592@zjhu.edu.cn

**Keywords:** *Procambarus clarkii*, copper, melatonin, oxidative stress, proteomic

## Abstract

Copper (Cu) contamination in aquatic environments induces oxidative stress and structural damage to crustaceans. This study investigated the protective effects and associated mechanisms of exogenous melatonin (MT) against Cu-induced toxicity in *Procambarus clarkii* using integrated physiological, histopathological, proteomic, and molecular analyses. MT supplementation enhanced antioxidant defense by elevating SOD, CAT, and T-AOC activities, while reducing MDA accumulation, with peak effects observed at 24 h. MT also restored endogenous melatonin levels and regulated phosphatase activity, thereby maintaining immune and metabolic homeostasis. Histopathology showed reduced hepatopancreatic damage, characterized by reduced epithelial vacuolization and preserved basement membrane integrity. Proteomics suggested that MT modulates a multilayered network associated with detoxification, redox balance, and cellular homeostasis. Pathway enrichment showed that Cu exposure dysregulated proteins involved in mitochondrial biogenesis, ABC transporters, membrane trafficking, and apoptosis. MT administration partially counteracted these alterations and was associated with the regulation of glutathione metabolism, as well as reduced enrichment of lysosome- and apoptosis-related pathways. Quantitative RT-PCR results were consistent with the proteomic data. Overall, MT partially alleviated Cu-induced toxicity and was associated with enhanced antioxidant defense, improved cellular homeostasis, and metabolic regulation. Our study provides new molecular insights and suggests its potential application for mitigating metal toxicity in aquaculture.

## 1. Introduction

Copper (Cu) contamination in aquatic ecosystems has become an increasing global environmental concern. Rapid industrialization, intensive mining activities, and agricultural discharge have exacerbated Cu accumulation in aquatic ecosystems, posing escalating risks to environmental and public health [1,2]. It has been reported that Cu concentrations in sediments from aquaculture ponds in Huzhou range from 10.50 to 98.20 mg kg^−1^, while those in Taihu Lake sediments range from 13.86 to 54.58 mg kg^−1^ [3,4]. Despite the implementation of environmental regulatory policies worldwide, the ecological hazards of Cu contamination in aquatic ecosystems remain profound, posing sustained and disproportionate threats to crustaceans, which play pivotal roles in trophic dynamics and ecosystem stability [5]. In addition, environmental factors, including pH, temperature, and microplastics, can affect the stress responses and bioaccumulation of copper in water [6,7].

Cu is an essential cofactor for numerous enzymatic systems in crustaceans and plays pivotal roles in physiological metabolism, immune regulation, and developmental processes [8,9,10]. Under homeostatic conditions, crustaceans maintain a dynamic equilibrium of intracellular Cu concentrations through sophisticated regulatory mechanisms involving coordinated processes of membrane carrier-mediated selective uptake, cytoplasmic chaperone protein-directed delivery, metal-binding protein chelation storage, and ATP-driven active efflux [11,12]. However, excessive Cu disrupts this balance, leading to the overproduction of reactive oxygen species (ROS) and severe oxidative stress, which are closely associated with increased ROS generation during oxidative phosphorylation [12,13]. Importantly, previous studies have demonstrated that the physiological responses of crustaceans to Cu exposure are both dose- and time-dependent [14,15]. Although various crustacean species can initially mitigate oxidative damage by regulating antioxidant enzymes, this compensatory mechanism gradually fails under sustained or high-intensity stress [12,15]. Therefore, identifying an appropriate time point at which stress responses are fully activated is critical for accurately evaluating the toxic effects of Cu and the efficacy of protective interventions. Oxidative damage is primarily characterized by lipid peroxidation, protein denaturation, and DNA fragmentation, ultimately leading to loss of cell membrane integrity, mitochondrial dysfunction, and activation of apoptotic pathways, which collectively compromise the resistance of organisms to pathogenic challenges [16,17]. Recent studies suggest that mitochondria are primary targets of Cu toxicity. As the core sites of oxidative phosphorylation, they are particularly vulnerable to metal-induced disturbances, which may disrupt electron transport and elevate ROS production [18]. Furthermore, Cu toxicity exhibits pronounced tissue specificity; gill tissues, which are in direct contact with the external environment, are the first to incur damage, whereas the hepatopancreas, the principal organ for detoxification and metabolism, undergoes more complex pathological alterations under chronic Cu exposure, including disruption of antioxidant enzyme homeostasis, suppression of immune function, and structural remodeling of tissues [11,15,19].

Melatonin (N-acetyl-5-methoxytryptamine, MT) is an endogenous indoleamine hormone synthesized from tryptophan via sequential hydroxylation and methylation reactions [20]. MT was initially discovered in the pineal glands of mammals and is ubiquitously distributed across diverse biological taxa [21,22,23]. As a signaling molecule with multiple biological functions, MT not only participates in circadian rhythm regulation but also exhibits potent antioxidant and immunomodulatory effects [24,25,26]. The indole ring and methoxy functional groups in its molecular structure enable direct scavenging of various ROS without enzymatic catalysis. Additionally, MT activates the endogenous antioxidant defense system by upregulating the expression and activity of key antioxidant enzymes such as superoxide dismutase (SOD), catalase (CAT), and glutathione peroxidase [27,28,29]. MT modulates key immune cell functions, including chemotaxis, phagocytosis, and apoptosis, thereby preserving immune homeostasis and enhancing the host defense capacity [30,31]. Exogenous MT exhibits a remarkable potential to safeguard aquatic animals from environmental stressors and to improve survival, immune competence, and growth performance across diverse species [32,33]. For example, in the Chinese mitten crab (*Eriocheir sinensis*), exogenous MT supplementation elevated eyestalk MT levels, enhanced hemolymphatic immune and antioxidant capacities, and significantly improved survival rates [34]. In Nile tilapia (*Oreochromis niloticus*), MT effectively mitigates silver nanoparticle toxicity and improves key biochemical parameters [35]. In crayfish (*Cherax destructor*), MT treatment significantly enhances antioxidant capacity, growth rates, and digestive enzyme activity, demonstrating promising application prospects for mitigating Cu exposure-induced biological damage [36]. However, MT efficacy varies with dose, exposure time, and species, and its molecular mechanism remains unclear, requiring further research to optimize application.

Red swamp crayfish (*Procambarus clarkii*), a freshwater crustacean of significant economic importance native to the southeastern United States, has a widespread global distribution [37]. Aquaculture production of this species in China reached 2.89 million tons in 2022, generating substantial economic value [38,39]. Because of its remarkable environmental adaptability, high reproductive potential, and broad habitat tolerance, *P. clarkii* is an ideal bioindicator species for assessing the toxicological impacts of metal pollution and for environmental monitoring [15,40]. Previous studies have shown that Cu exposure markedly impairs the biological performance of *P. clarkii*, leading to physiological dysfunction, reduced survival, and inhibited growth and development [6,16]. High Cu exposure not only suppresses immune responsiveness but also disrupts fundamental metabolic processes, leading to functional impairment of key antioxidant enzyme systems in hepatopancreatic tissues [11,15]. Previous studies on *P. clarkii* demonstrated that exogenous MT supplementation significantly enhances juvenile weight gain, specific growth rate, and overall survival rate [41,42,43]. MT can enhance the catalytic activity of lysozyme and alkaline phosphatase in the hemolymph and activate transcription of circadian rhythm-regulating genes in the hepatopancreas [41,43]. MT intervention also enhances endogenous antioxidant defense systems, stimulates circulating hemocyte proliferation, activates cellular phagocytic function, and upregulates the expression of antioxidant- and immune-related transcription factors [42,43]. Transcriptomic analysis has revealed that MT exerts immune-enhancing effects primarily by regulating the gene expression patterns of key immune networks, including Toll-like receptor signaling pathways, protease inhibitor systems, and C-type lectin recognition mechanisms [41]. However, the precise molecular mechanisms by which MT mitigates Cu-induced oxidative stress and hepatopancreatic injury, particularly from a proteomic perspective, remain largely unclear in *P. clarkii*.

In the present study, biochemical and histopathological analyses were used to evaluate the ameliorative effects of MT on Cu-induced oxidative damage and hepatopancreatic lesions in *P. clarkii*. Subsequently, quantitative proteomic profiling was performed to identify differentially expressed proteins (DEPs) under Cu exposure, MT treatment, and combined conditions, followed by functional enrichment analyses to identify the key regulatory pathways. Based on the oxidative toxicity of Cu and the protective properties of MT, we hypothesized that Cu exposure may induce oxidative stress and hepatopancreatic injury in *P. clarkii*, potentially associated with mitochondrial dysfunction. We further hypothesized that exogenous MT could alleviate these effects by improving antioxidant and immune-related responses and modulating stress-related molecular pathways. By integrating physiological, histopathological, proteomic, and molecular validation data, we provide mechanistic insights into how exogenous MT may mitigate Cu toxicity in *P. clarkii*. Collectively, these findings provide novel insights into metal toxicology in crustaceans and offer a theoretical basis for developing MT-based bioprotective strategies for aquaculture systems.

## 2. Results

### 2.1. Exogenous MT Enhanced Antioxidant Enzyme Activities and Mitigated Cu-Induced Lipid Peroxidation in the Hepatopancreas of P. clarkii

**Figure 1:**
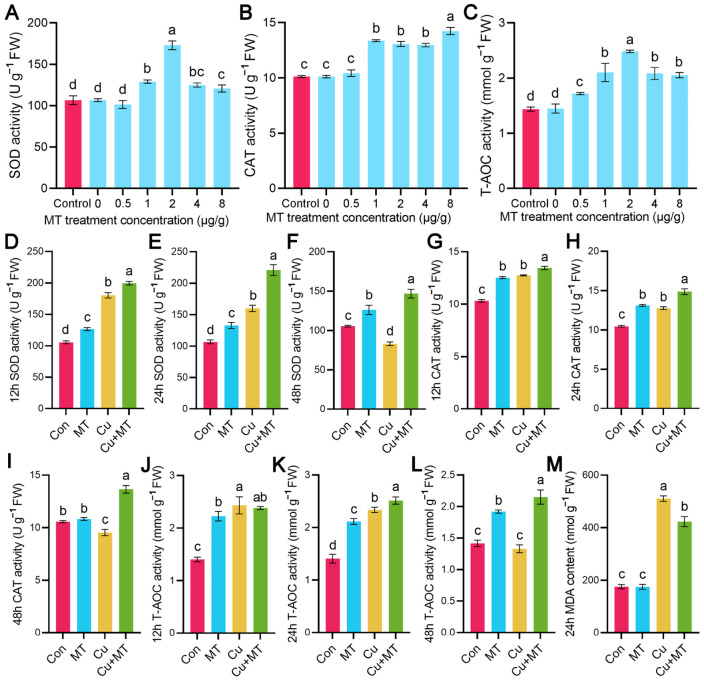
Effects of MT on antioxidant responses in the hepatopancreas of *P. clarkii*. (**A**–**C**) Antioxidant enzyme activities among seven groups (control and 0–8 µg g^−1^ MT) at 24 h: (**A**) SOD, (**B**) CAT, and (**C**) T-AOC. (**D**–**F**) SOD activity at 12, 24, and 48 h under different treatments (Con, MT, Cu, and Cu+MT). (**G**–**I**) CAT activity at 12, 24, and 48 h. (**J**–**L**) T-AOC at 12, 24, and 48 h. (**M**) MDA content at 24 h. Data are presented as mean ± standard deviation (SD) (*n* = 3). Different lowercase letters above bars indicate statistically significant differences among treatment groups (*p* < 0.05, one-way ANOVA followed by Tukey’s test).

Seven treatment groups were used to investigate the dose-dependent effects of exogenous MT on antioxidant enzyme activities in the hepatopancreas of *P. clarkii*, including a control and six injection groups (0, 0.5, 1, 2, 4, and 8 μg g^−1^). No significant differences were observed between the 0 μg g^−1^ group and the control. At 24 h, treatment with 1 μg g^−1^ MT significantly enhanced the activities of SOD, CAT, and T-AOC by 21.0%, 32.1%, and 46.2%, respectively, compared with the control (Figure 1A–C). Collectively, these findings established 1 µg g^−1^ MT as the minimal effective dose for enhancing antioxidant enzyme activities. All biochemical analyses were performed with three biological replicates (*n* = 3). This concentration was therefore adopted for subsequent Cu stress mitigation assays. It has been shown that Cu stress can induce a wide range of antioxidant enzyme activities in the hepatopancreas of *P. clarkii* [11,15,44].

Based on the results of melatonin concentration screening, four experimental groups were established: Con (control group), MT (melatonin-treated group, 1 μg g^−1^), Cu (Cu-exposed group, 6 mg L^−1^), and Cu+MT (combined treatment group). As expected, compared with the Con group, SOD, CAT, and T-AOC activities showed a time-dependent trend under Cu exposure, with consistent patterns observed at 12, 24, and 48 h. At 12 h (Figure 1D,G,J), antioxidant enzyme activities were rapidly induced, with SOD and T-AOC showing marked increases, indicating the early activation of oxidative stress responses. However, the differences between the Cu and Cu+MT groups at this stage were relatively limited for certain indices, suggesting that the regulatory effect of MT had not yet been fully established. At 24 h, compared with the Con group, SOD, CAT, and T-AOC activities increased by 49.9%, 21.9%, and 65.9%, respectively, in the Cu-treated group (Figure 1E,H,K). Remarkably, co-treatment with Cu and MT further enhanced antioxidant responses, with SOD, CAT, and T-AOC activities elevated by 107.8%, 42.1%, and 78.6%, respectively, relative to those in the Con group. At this time point, the Cu+MT group consistently exhibited the highest enzyme activities, indicating that both Cu-induced oxidative stress and MT-mediated antioxidant enhancement were most evident at 24 h. At 48 h (Figure 1F,I,L), antioxidant enzyme activities in the Cu-treated group declined markedly, with SOD, CAT, and T-AOC levels decreasing to or even below those of the Con group. In contrast, MT treatment partially maintained enzyme activities, indicating its sustained protective role during later stages of stress. Further analysis of lipid peroxidation revealed that, compared with the Con group, MDA content increased by 190.8% in the Cu-treated group, whereas the Cu+MT treatment resulted in a lower increase of 140.7% (Figure 1M), indicating an alleviating role of MT in lipid peroxidation under Cu stress. Taken together, these results demonstrate a clear time-dependent pattern of antioxidant responses, with 24 h representing the optimal time point at which oxidative stress is fully activated while MT-mediated protection is most pronounced.

### 2.2. Exogenous MT Attenuated Cu-Induced Histopathological Damage, Modulated Phosphatase Activities, and Restored Endogenous MT Levels

**Figure 2:**
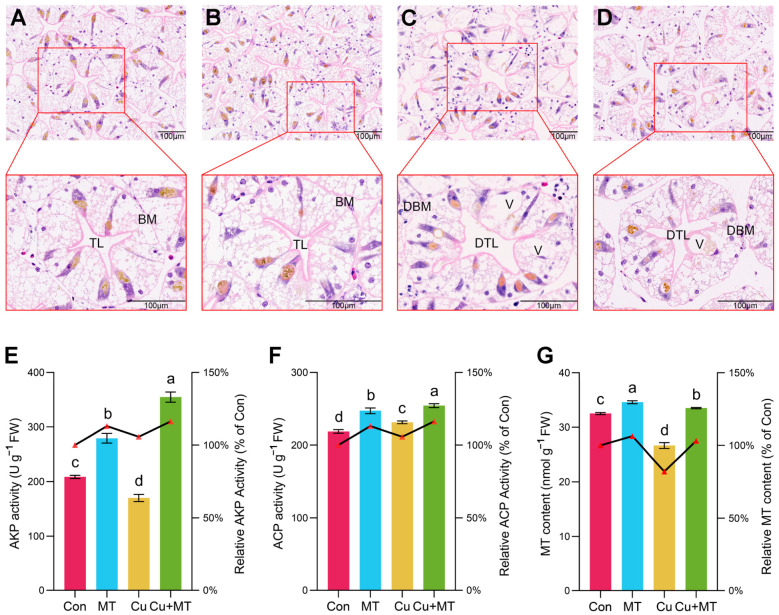
Effects of MT on histopathological alterations, phosphatase activities, and endogenous MT content in the hepatopancreas of *P. clarkii* after 24 h exposure. (**A**–**D**) Histopathological sections of the hepatopancreas under different treatments: (**A**) Con, (**B**) MT, (**C**) Cu, and (**D**) Cu+MT. Key pathological features are indicated as follows: basement membrane (BM), tubule lumen (TL), dilated tubule lumen (DTL), vacuolization (V), and disintegrated basement membrane (DBM). Sections were stained with hematoxylin and eosin (H&E) and imaged at 100× magnification. Histological observations are representative of three biological replicates. (**E**–**G**) Changes in phosphatase activities and endogenous MT levels: (**E**) alkaline phosphatase (AKP) activity, (**F**) acid phosphatase (ACP) activity, and (**G**) endogenous MT content. Bars indicate absolute values (left axis), whereas lines represent the relative percentage of Con (right axis). Data in (**E**–**G**) are presented as mean ± standard deviation (SD) (*n* = 3). Different lowercase letters above bars indicate statistically significant differences among treatment groups (*p* < 0.05, one-way ANOVA followed by Tukey’s test).

To further evaluate the protective role of MT against Cu-induced histopathological damage in *P. clarkii*, histopathological analysis was conducted. These results showed that the hepatopancreatic tubules in the Con group displayed intact architecture, with stellate lumens, orderly arranged tissue, and no appreciable vacuolization (Figure 2A). A similar histological profile was observed in the MT group (Figure 2B). In contrast, Cu exposure induced severe damage, including extensive epithelial vacuolization, basement membrane disintegration, and dilated tubular lumen formation (Figure 2C). Although these pathological changes were still observed in the Cu+MT group, their severity was markedly reduced, with most cells maintaining relatively intact morphology compared with those in the Cu-treated group (Figure 2D). Collectively, these results demonstrate that MT exerts a protective effect against Cu-induced hepatopancreatic injury in *P. clarkii*. Further analysis of phosphatase activity showed that, relative to the Con group, Cu exposure reduced AKP activity by 18.6% (Figure 2E) but slightly increased ACP activity by 5.6% (Figure 2F). Co-treatment with MT significantly reversed these responses, elevating AKP and ACP activities by 70.0% and 16.2%, respectively, above the control levels (Figure 2E,F). Concurrently, endogenous MT content decreased by 18.0% under Cu stress, whereas Cu+MT co-treatment restored MT levels, resulting in a 25.7% increase relative to that in the Cu-treated group (Figure 2G). These observations are consistent with those of previous studies that showed that exogenous MT supplementation can significantly enhance AKP and ACP activities in aquatic animals [45,46]. Taken together, these findings indicate that exogenous MT alleviates Cu-induced hepatopancreatic damage in *P. clarkii* by restoring endogenous MT levels and modulating phosphatase activity.

### 2.3. Proteomic Analysis of Hepatopancreas in P. clarkii Under Cu Stress and MT Treatment

**Figure 3:**
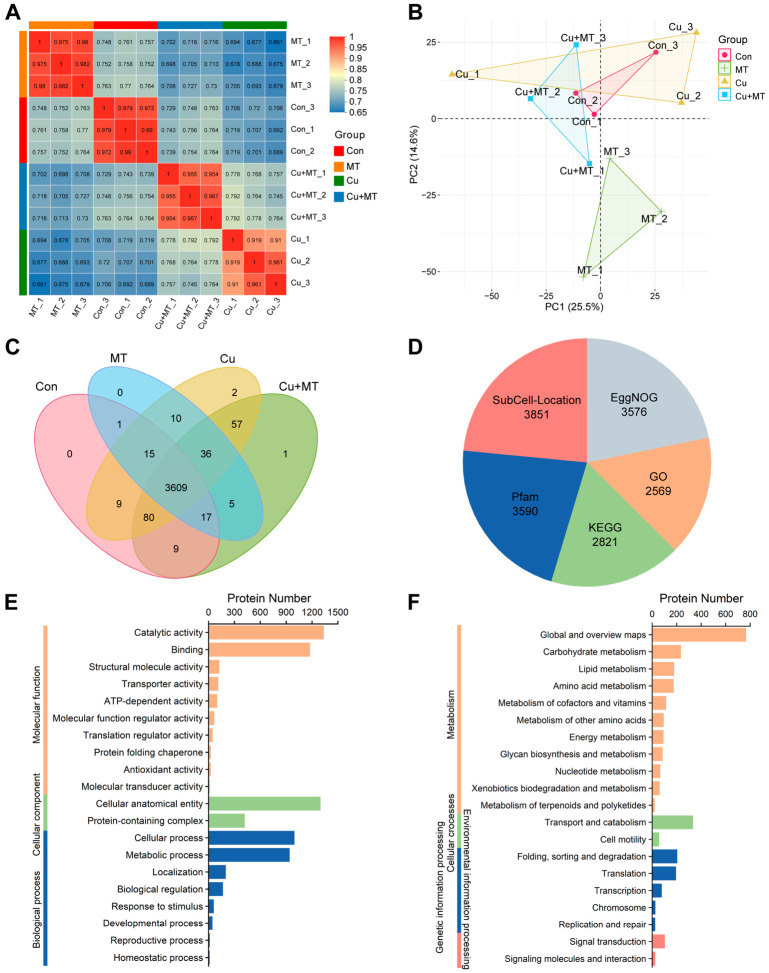
Proteomic analysis of the hepatopancreas in *P. clarkii* under Cu stress and MT treatment. (**A**) Protein correlation analysis among samples. (**B**) Principal component analysis (PCA) of identified proteins among different treatment groups. (**C**) Venn diagram illustrating the overlap of identified proteins across different treatment groups. (**D**) Number of proteins annotated via different databases, including Pfam, EggNOG, GO, KEGG, and SubCell-Location. (**E**) Classification information of annotated GO terms for proteins. (**F**) Classification information of annotated KEGG terms for proteins. Proteomic analysis was performed using three biological replicates per group (*n* = 3).

To elucidate the molecular mechanisms underlying MT-mediated alleviation of Cu stress in *P. clarkii*, a comprehensive proteomic analysis was performed on hepatopancreas samples. Peptide lengths were mainly distributed between 7 and 22 amino acids, with the quantity initially increasing and then decreasing with length (Figure S1A), and the protein abundance gradually decreased with increasing molecular weight (Figure S1B). The proteomic analysis identified 28,253 peptides and 3892 protein groups. After filtering for missing values (removing proteins with a missing rate > 90%), 3851 protein groups were retained for subsequent analyses (Table S2). Among these, 58% exhibited a sequence coverage exceeding 10% (Figure S1C). Pearson’s correlation coefficient between samples within the same group exceeded 0.91, indicating good experimental reproducibility (Figure 3A). Principal component analysis (PCA) showed that the MT group was clearly separated from the other groups (Figure 3B). Venn analysis confirmed the presence of 3609 common proteins across all groups, providing a consistent background for comparative analysis (Figure 3C).

Functional annotation using Pfam, EggNOG, GO, KEGG, and SubCell-Location databases was conducted to provide a functional framework for downstream interpretation. Among the 3851 retained proteins, 2569 were assigned GO annotations (Figure 3D). The dominant terms were “Catalytic activity,” “Binding,” and “Structural molecule activity” for molecular function; “Cellular process,” “Metabolic process,” and “Localization” for biological process; and “Cellular anatomical entity” and “Protein-containing complex” for cellular component (Figure 3E). KEGG mapping classified proteins into four categories (Figure 3F), with “Metabolism” being dominant with 1889 proteins, characterized by “Global and overview maps,” “Carbohydrate metabolism,” “Lipid metabolism,” and “Amino-acid metabolism.” Other major sub-categories included “Transport and catabolism” within cellular processes, “Folding, sorting and degradation” and “Translation” within genetic information processing, and “Signal transduction” containing 103 proteins within environmental information processing. Collectively, these results indicate that the adaptive responses of the *P. clarkii* hepatopancreas to Cu stress and MT intervention involve a wide range of metabolic and regulatory pathways, and provide a basis for subsequent analysis of MT-responsive pathways.

### 2.4. Proteomic Response of P. clarkii to Cu Stress and MT Treatment

**Figure 4:**
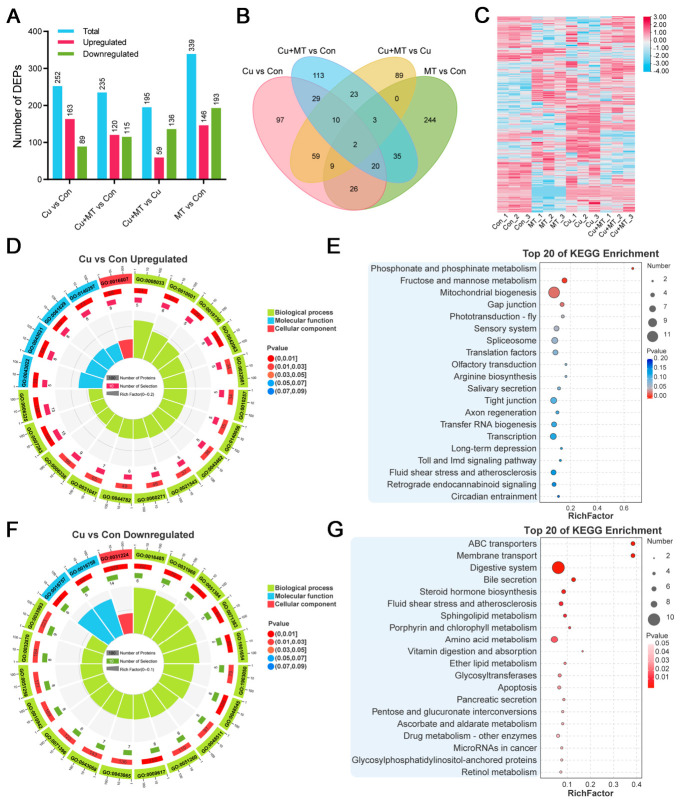
Screening of DEPs in *P. clarkii* treated with Cu and/or MT. (**A**) Bar plot showing the number of DEPs in the differential comparison combinations. Red and green represent upregulated and downregulated proteins, respectively. Numbers above bars indicate the specific count of proteins. (**B**) Venn diagram illustrating the overlap of DEPs in the differential comparison combinations. (**C**) Hierarchical clustering heatmap of DEPs identified across the four treatment groups. The color gradient from blue to red indicates increasing protein expression levels. (**D**–**G**) Functional enrichment analysis of DEPs in the Cu vs. Con comparison: (**D**) GO enrichment circle plot of upregulated DEPs, (**E**) Top 20 KEGG pathways enriched in upregulated DEPs, (**F**) GO enrichment circle plot of downregulated DEPs, and (**G**) Top 20 KEGG pathways enriched in downregulated DEPs.

To elucidate the mechanism by which exogenous MT alleviated Cu stress in *P. clarkii*, we investigated the DEPs between different treatments (Figures S2–S5). In total, 759 DEPs were identified through pairwise comparisons across the four treatment groups (Figure 4A). A Venn diagram illustrates the overlap of DEPs among the differential comparison groups, with two DEPs being common across all four comparisons (Figure 4B). Furthermore, a hierarchical clustering heatmap provides a global overview of DEP profiles across all treatments (Figure 4C). Relative to the Con group, the Cu treatment resulted in 163 upregulated and 89 downregulated DEPs (Figure 4A). Functional analysis of the upregulated DEPs revealed significant GO enrichment in biological processes such as “Gliogenesis,” “tRNA processing,” and “Antimicrobial humoral response,” whereas molecular functions were predominantly associated with “ribonucleoprotein complex binding” and “ribosome binding” (Figure 4D).

KEGG pathway analysis of the upregulated DEPs highlighted significant enrichment in pathways including “Phosphonate and phosphinate metabolism,” “Fructose and mannose metabolism,” “Mitochondrial biogenesis,” and “Gap junction” (Figure 4E). These findings suggest that these signaling pathways may be activated in the hepatopancreas of *P. clarkii* as an adaptive response to Cu stress. Conversely, for the downregulated DEPs, GO analysis revealed enrichment in processes including “Response to corticosteroid” and “Positive regulation of apoptotic process” (Figure 4F). Correspondingly, KEGG analysis indicated significant enrichment in pathways such as “ABC transporters,” “Membrane transport,” and “Apoptosis” (Figure 4G). These observations suggest that Cu exposure may disturb membrane transport processes and promote apoptosis-related signaling, possibly in association with oxidative stress and mitochondrial injury. Cu exposure has been widely reported to directly target mitochondria, impairing mitochondrial integrity and redox homeostasis in aquatic animals [47,48]. Collectively, these results indicate that Cu stress affects multiple cellular processes related to mitochondrial regulation, membrane transport, and apoptosis in the hepatopancreas of *P. clarkii*.

### 2.5. Identification of DEPs Related to the Alleviation of Cu Stress by Exogenous MT in P. clarkii

**Figure 5:**
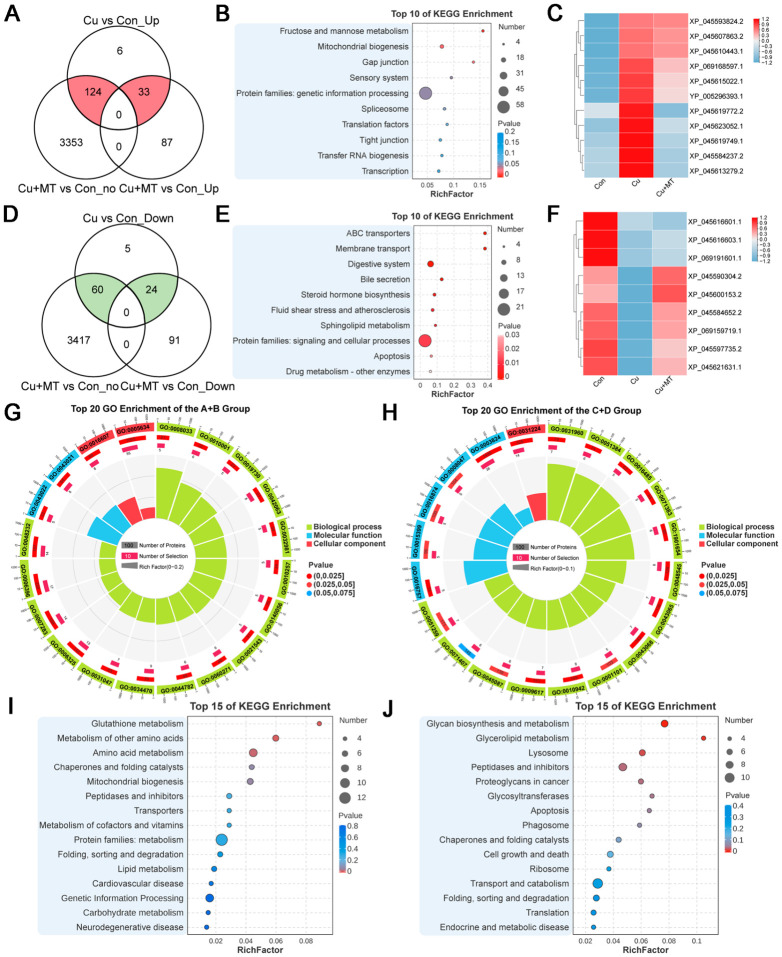
Identification of DEPs and MT-specific regulatory proteins involved in the alleviation of Cu stress by exogenous MT in *P. clarkii*. (**A**) Venn diagram of Cu-induced protein upregulation regulated by MT. (**B**) Top 10 KEGG enrichment of DEPs in the A + B groups. (**C**) Heatmap illustrating the relative expression levels of DEPs in the “Mitochondrial biogenesis” pathway across different sample groups. (**D**) Venn diagram of MT-induced protein downregulation in Cu-exposed *P. clarkii*. (**E**) Top 10 KEGG enrichment of DEPs in the C + D group. (**F**) Heatmap of DEPs in ABC transporters, membrane transport, and apoptosis pathways. (**G**) Top 20 GO enrichment of DEPs in the A + B group. (**H**) Top 20 GO enrichment of DEPs in the C + D group. (**I**) Top 15 KEGG pathways enriched in MT-specific upregulated proteins. (**J**) Top 15 KEGG pathways enriched in MT-specific downregulated proteins.

To further elucidate the role of MT in mitigating Cu stress in *P. clarkii*, we analyzed the DEPs across the treatment groups and identified 241 proteins involved in stress alleviation. Specifically, we focused on 124 proteins (group A) in the overlap of upregulated proteins in the Cu vs. Con group and non-regulated proteins in the Cu+MT vs. Con group, as well as 33 proteins (group B) in the overlap of upregulated proteins in both the Cu vs. Con and Cu+MT vs. Con groups (Figure 5A). Similarly, we examined 60 proteins (group C) in the overlap of downregulated proteins in the Cu vs. Con group and non-regulated proteins in the Cu+MT vs. Con group, and 24 proteins (group D) in the overlap of downregulated proteins in both the Cu vs. Con and Cu+MT vs. Con groups (Figure 5D). KEGG enrichment analysis of the combined A + B group highlighted “Fructose and mannose metabolism,” “Mitochondrial biogenesis,” and “Gap junction” (Figure 5B), with heatmaps confirming that MT treatment markedly counteracted Cu-induced alterations in the “Mitochondrial biogenesis” pathway (Figure 5C). Conversely, the C + D group was enriched in 14 pathways, including “ABC transporters,” “Membrane transport,” and “Apoptosis” (Figure 5E), where MT similarly mitigated expression changes (Figure 5F). Thus, MT primarily alleviates Cu stress via “Mitochondrial biogenesis,” “ABC transporters,” “Membrane transport,” and “Apoptosis” pathways. These findings are consistent with reports that MT maintains cellular homeostasis by enhancing mitochondrial efficiency and regulating apoptotic signaling under environmental stress [49,50].

To gain deeper insight into the functional roles of DEPs in MT-mediated Cu stress alleviation, GO enrichment analysis was conducted separately for the A + B and C + D groups, as shown in Figure 5G,H. In the A + B group (Figure 5G), DEPs were primarily enriched in biological process categories involving RNA metabolism and processing pathways such as “tRNA processing” and “Regulatory ncRNA-mediated gene silencing.” Significant enrichments were also found in mitochondrial function, including “Mitochondrial respiratory chain complex I assembly,” as well as immune-related terms such as “Antimicrobial humoral response.” For the cellular component, enrichment was predominantly observed in “Nuclear speck” and “Nucleus.” Regarding molecular function, notable enrichments included “ribonucleoprotein complex binding” and “ribosome binding.” In the C + D group (Figure 5H), DEPs exhibited distinct enrichment patterns in hormone response terms, alongside protein regulation- and apoptosis-related processes. Specifically, significant enrichment was identified in the “Response to corticosteroid,” “Response to steroid hormone,” “Protein processing,” “Positive regulation of apoptotic process,” and “Positive regulation of programmed cell death.” Furthermore, “Response to bacterium” and “Innate immune response” terms showed significant enrichment, whereas molecular functions encompassed “Glycosyltransferase activity” and “Primary active transmembrane transporter activity.” These results collectively suggest that MT modulates diverse biological processes in *P. clarkii* to achieve comprehensive protection against Cu-induced toxicity.

Further analysis revealed that 87 proteins were significantly upregulated and 91 proteins were downregulated by Cu+MT treatment but were not induced by Cu stress alone (Figure 5I,J). KEGG enrichment analysis indicated that the upregulated proteins were significantly enriched (*p* < 0.05) in three pathways, including “Glutathione metabolism,” “Metabolism of other amino acids,” and “Amino acid metabolism” (Figure 5I). These findings are consistent with previous studies showing that MT can stimulate the synthesis of endogenous antioxidants and promote amino acid cycling, thereby providing essential precursors for stress-response proteins [46,51]. In contrast, the downregulated proteins were significantly enriched in seven pathways, including “Glycan biosynthesis and metabolism,” “Glycerolipid metabolism,” “Lysosome,” “Peptidases and inhibitors,” “Proteoglycans in cancer,” “Glycosyltransferases,” and “Apoptosis” (Figure 5J). In line with previous research, the inhibition of “Lysosome” and “Apoptosis” pathways underscores the capacity of MT to stabilize cellular integrity by preventing excessive degradation and programmed cell death during metal exposure [52]. Collectively, these findings suggest that exogenous MT confers additional alleviation capacity through the specific regulation of proteins, thereby enhancing the capacity of *P. clarkii* to alleviate Cu toxicity.

### 2.6. qRT-PCR Validation of DEPs

**Figure 6:**
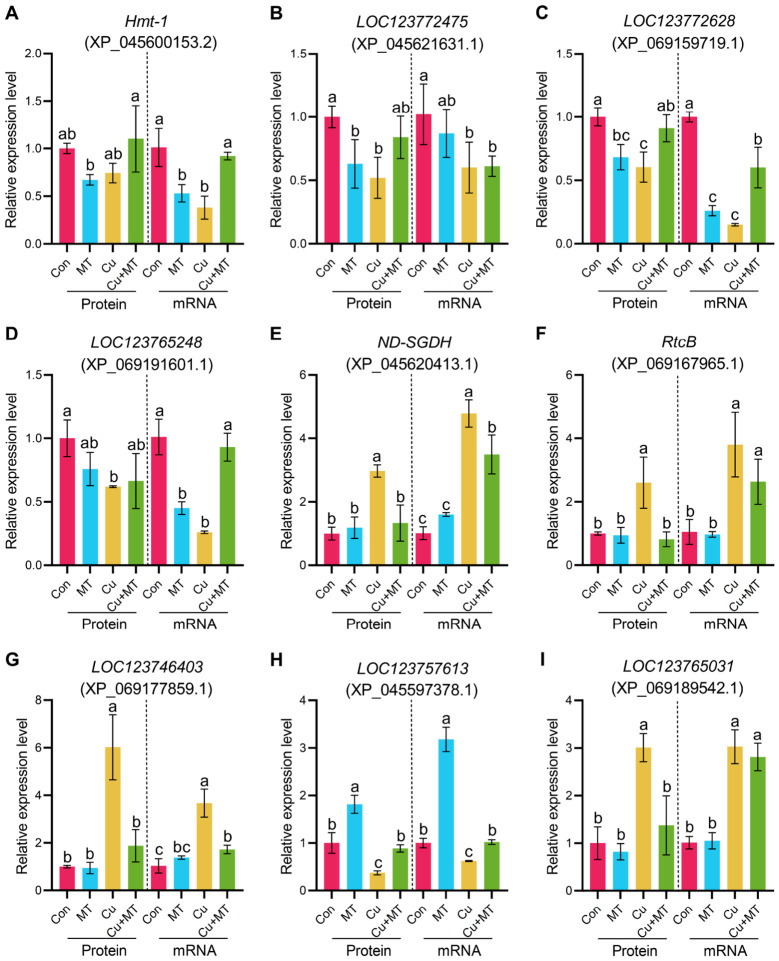
Transcriptional validation of selected proteins in the hepatopancreas of *P. clarkii*. (**A**–**I**) Verification of the genes at the transcriptional level using qRT-PCR in comparison with protein expression at the translational level. The transcriptional levels of genes corresponding to nine selected proteins were analyzed using *18S rRNA* as an internal control. Data are expressed as mean ± standard deviation (*n* = 3). Different lowercase letters above bars denote statistically significant differences among treatment groups (*p* < 0.05, one-way ANOVA followed by post hoc test).

To verify the reliability of the proteomic profiles and further validate the expression trends of the identified DEPs in the ABC transporter pathway, nine representative genes, including *Hmt-1*, *ND-SGDH*, and *RtcB*, were selected for qRT-PCR analysis (Figure 6A–I). The results revealed a high degree of concordance between the mRNA expression levels and protein abundance patterns across all treatment groups. Specifically, proteins downregulated by Cu stress but significantly restored by MT supplementation, such as *Hmt-1* (Figure 6A) and *LOC123772628* (Figure 6C), exhibited synchronous transcriptional reductions and recoveries. Conversely, Cu-induced proteins, such as *ND-SGDH* (Figure 6E) and *RtcB* (Figure 6F), showed parallel increases in gene expression, which were subsequently mitigated in the Cu+MT group. This strong correlation between mRNA transcription and protein translation underscores the robustness of our proteomic dataset, suggesting that the MT-mediated response to Cu stress is consistently regulated at both the transcriptional and translational levels.

## 3. Discussion

### 3.1. MT Mitigates Cu-Induced Physiological Dysfunction and Histopathological Damage

Rapid industrialization over the past few decades has exacerbated metal pollution, with Cu posing a severe threat to aquatic ecosystems [1,2]. Although Cu is an essential trace element, excess Cu can induce significant physiological dysfunction in crustaceans, leading to oxidative stress, lipid peroxidation, and tissue damage [11,12]. These effects manifest as ROS overproduction, which disrupts cellular integrity and impairs metabolic functions [19,53]. In the present study, Cu exposure caused severe oxidative damage in *P. clarkii*, as evidenced by a sharp increase in MDA content (Figure 1M) and significant histopathological alterations in the hepatopancreas, including epithelial vacuolization and tubular dilation (Figure 2C). These findings highlight the detrimental effects of Cu on the structural and functional integrity of the hepatopancreas, the primary detoxification organ in crustaceans.

However, application of exogenous MT significantly mitigated these adverse effects. MT, a potent antioxidant, is pivotal in maintaining cellular redox homeostasis and regulating the immune defense system [34,36]. In this study, MT treatment significantly enhanced key physiological parameters, including the activities of the antioxidant enzymes SOD, CAT, and T-AOC at 24 h (Figure 1A–C) and phosphatases AKP and ACP (Figure 2E,F). Notably, Cu exposure significantly suppressed endogenous MT levels, likely by inhibiting biosynthetic enzymes. However, exogenous supplementation effectively restored these levels (Figure 2G) and MT treatment markedly reduced lipid peroxidation (Figure 1M). However, the reduction in MDA was relatively limited, and its level remained higher than that in the Con, suggesting that MT provides only partial protection against Cu-induced lipid peroxidation. This may be due to the strong oxidative stress induced by Cu and the limited capacity of MT to fully reverse membrane damage. Furthermore, antioxidant responses exhibited distinct time-dependent patterns (Figure 1D–L). In the Cu-treated group, enzyme activities declined markedly at later stages, indicating a progressive impairment of the antioxidant system. In contrast, MT treatment sustained higher activity levels over time, with the differences among groups becoming most pronounced at 24 h. These observations suggest that 24 h represents a critical time window during which oxidative stress is sufficiently activated and MT-mediated protection is most evident. Consistent with previous studies [36,42,54], our results suggest that MT effectively counteracts Cu-induced stress by boosting antioxidant defense. Furthermore, histopathological analysis confirmed that MT preserved the hepatopancreatic architecture, reduced vacuolization, and maintained cell morphology (Figure 2D). This restoration of tissue integrity and enzymatic function underscores the critical role of MT in facilitating the recovery of *P. clarkii* under metal stress, likely by scavenging ROS and preventing oxidative injury to cellular components [45,55].

### 3.2. MT Restores Cellular Homeostasis via Transport and Mitochondrial Pathways

In this study, proteomics of *P. clarkii* hepatopancreas (Figure 5) revealed that MT-mediated alleviation of Cu stress may involve the modulation of key pathways, including ABC transporters, membrane transport, and mitochondrial biogenesis. The ABC transporter family and membrane transport systems play a key role in metal ion transport and sequestration, utilizing ATP to actively compartmentalize metal ions [10,12,56]. Cu stress typically impairs these transport mechanisms, leading to intracellular Cu accumulation and toxicity [12,19]. Our data indicate that MT treatment may partially restore the expression of proteins associated with these pathways (Figure 5C,F), suggesting a potential role in Cu handling and ion homeostasis.

Furthermore, Cu exposure disrupts the mitochondrial electron transport chain, causing mitochondrial dysfunction and energy deficits [57,58]. Mitochondria are not only energy powerhouses but also the primary sites of ROS generation [59,60]. The proteomics results showed that MT upregulates proteins involved in mitochondrial biogenesis (Figure 5C), suggesting a compensatory mechanism by which MT may contribute to mitochondrial repair or regeneration to counteract Cu-induced respiratory inhibition. MT may contribute to the preservation of energy metabolism and cellular homeostasis by modulating proteins associated with mitochondrial biogenesis and membrane transport, effectively preventing the damage cascade initiated by Cu accumulation.

### 3.3. MT Enhances Detoxification Through Glutathione Metabolism and Lysosomal Regulation

This study further revealed the specific regulation of glutathione metabolism and lysosome pathways, highlighting a dual mechanism of protection. GSH is a fundamental component of the nonenzymatic antioxidant system and is crucial for chelating metal ions and scavenging free radicals [61,62,63]. Cu stress often leads to GSH depletion owing to the formation of Cu-GSH complexes [64]. In this study, proteins related to glutathione metabolism were significantly upregulated in the Cu+MT group (Figure 5I). We hypothesized that MT may stimulate the synthesis of endogenous GSH and related amino acids, thereby providing sufficient substrate to sequester free Cu^2+^ and reduce its cytotoxicity [46,65]. This upregulation may enhance the capacity of the organism to cope with metal stress.

Conversely, the lysosome and apoptosis pathways were significantly downregulated by MT treatment (Figure 5J). Lysosomes serve as the final repository of sequestered metals; however, excessive accumulation can compromise lysosomal membrane stability, leading to the leakage of cathepsins and the triggering of cell death [64,66,67]. Downregulation of lysosome-related proteins suggests that MT may help preserve lysosomal stability and limit downstream cell-death signaling under Cu stress [68,69]. MT may exert coordinated protective effects by enhancing glutathione-related detoxification and attenuating lysosome-associated cell death. These findings provide a comprehensive understanding of how MT orchestrates metabolic and regulatory networks to enhance the resilience of *P. clarkii* to Cu toxicity.

### 3.4. MT Orchestrates a Coordinated Regulatory Network to Mitigate Cu Toxicity

**Figure 7:**
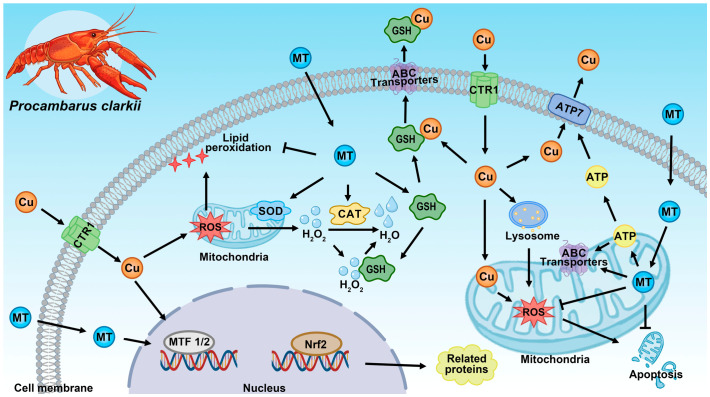
Schematic model illustrating the potential mechanisms underlying MT-mediated alleviation of Cu-induced hepatopancreatic toxicity in *P. clarkii*. CTR1: copper transporter 1; ABC transporters: ATP-binding cassette transporters; MT: melatonin; ROS: reactive oxygen species; GSH: glutathione; SOD: superoxide dismutase; CAT: catalase; ATP: adenosine triphosphate; ATP7: ATPase copper transporting; MTF1/2: metal-response element-binding transcription factor 1/2; Nrf2: nuclear factor erythroid 2-related factor 2. Arrows indicate pathway activation, and T-bars indicate repression.

Based on the physiological, histopathological, and proteomic results, together with previous evidence [12,36,41,70,71,72], we propose a putative regulatory network for MT-mediated mitigation of Cu toxicity in *P. clarkii* (Figure 7). In this model, Cu exposure may disturb intracellular metal homeostasis and trigger oxidative stress, leading to lipid peroxidation, organelle dysfunction, and apoptosis-related responses [12]. Mitochondria appear to be an important convergence point in this process [72]. The enrichment of mitochondrial biogenesis-related proteins suggests that Cu stress may affect mitochondrial maintenance, whereas MT treatment may help restore mitochondrial homeostasis and support energy metabolism [71]. In addition, MT may contribute to Cu stress alleviation by coordinating transport and antioxidant responses. The enrichment of ABC transporters and membrane transport pathways suggests a possible role of MT in modulating ion and metal handling, thereby influencing intracellular Cu distribution and homeostasis rather than directly blocking its accumulation [50]. Meanwhile, the observed increased SOD, CAT, and T-AOC activities, together with reduced MDA levels, indicate that MT strengthens antioxidant defense and limits lipid peroxidation [36,65]. The activation of glutathione metabolism may further support ROS detoxification and metal buffering [12,71]. MT also appears to influence lysosome- and apoptosis-related pathways, suggesting a potential role in maintaining organelle integrity and reducing excessive cell-death signaling. These effects may act in concert with mitochondrial regulation, collectively contributing to cellular stabilization under Cu stress. Collectively, MT may alleviate Cu-induced hepatopancreatic toxicity through a coordinated network involving mitochondrial regulation, redox balance, transport homeostasis, glutathione-dependent detoxification, and suppression of apoptosis. Nevertheless, further functional assays, such as mitochondrial membrane potential, ATP production, mitochondrial ROS, Cu distribution, and transporter activity, are needed to validate this proposed mechanism.

## 4. Materials and Methods

### 4.1. Animal Materials

Healthy *P. clarkii* individuals (body length: 9.0 ± 0.5 cm; body weight: 18.0 ± 1.0 g) were obtained from an aquaculture facility in Huzhou, Zhejiang Province, China, and immediately transported to the laboratory for experimentation. Before experimentation, *P. clarkii* individuals were acclimated to laboratory conditions for 7 days under controlled environmental parameters, with water temperature maintained at 25.0 ± 1.0 °C, pH at 7.0 ± 0.2, and dissolved oxygen levels exceeding 6.8 ± 0.2 mg L^−1^ as described previously [7]. During the acclimation period, *P. clarkii* were fed a commercially formulated diet (Tongwei Feed Co., Ltd., Wuxi, China) at 2.0% of their body weight, twice daily. Uneaten feed and fecal residues were removed within 2 h of feeding, and feeding was suspended 12 h before the experimental treatments. The formal experimental phase was conducted using partitioned aquaculture systems composed of plastic aquaria (56 × 41 × 23 cm). Each aquarium was filled with 15 L of dechlorinated tap water and contained 15 individuals, each separated by isolation plates to prevent physical interaction. One-third of the water volume was renewed daily, and key water quality parameters were routinely monitored throughout the experiment [73,74]. The experiments were conducted in accordance with the Guidelines for Ethical Review of Laboratory Animal Welfare issued by the National Health Commission of China. The Animal Ethics Committee of Huzhou Normal University approved the experimental protocols.

### 4.2. Experimental Design and Cu Exposure

To determine the optimal dosage of MT, *P. clarkii* were randomly divided into seven groups: an untreated control group and six groups injected with 0, 0.5, 1, 2, 4, and 8 μg g^−1^ body weight of MT, respectively. These doses were selected based on previous studies on MT administration in crustaceans and designed as a gradient to assess dose-dependent responses [75]. The MT solution (Sigma-Aldrich, Darmstadt, Germany) was diluted in physiological saline (0.9% NaCl) and administered intramuscularly to the second dorsal abdominal segment using a microsyringe [76,77]. Hepatopancreatic tissues were collected 12, 24, and 48 h after injection. Each treatment included three biological replicates (*n* = 3), and each biological replicate consisted of pooled hepatopancreatic tissues from five individual crayfish. Based on preliminary experiments, four treatment groups were established: a control group without any supplementation (Con), an MT-treated group (MT, 1 μg g^−1^ body weight), a Cu stress group (Cu, 6 mg L^−1^ CuSO_4_), and a combined treatment group (Cu+MT, 6 mg L^−1^ CuSO_4_ + 1 μg g^−1^ body weight of MT). The Cu stress solution was prepared from copper(II) sulfate pentahydrate (CuSO_4_·5H_2_O; Sigma-Aldrich). The exposure concentration (6 mg L^−1^) was selected based on previously reported 96 h LC_50_ values for *P. clarkii* [15,44], corresponding to one-fifth of the LC_50_. This sublethal concentration was chosen to induce measurable physiological and biochemical responses without causing excessive mortality. Cu concentrations in the test medium were regularly monitored using a portable copper ion meter and adjusted to maintain nominal levels. In the combined treatment group, MT and Cu were administered simultaneously, and hepatopancreatic tissues were collected 12, 24, and 48 h after exposure. The samples were immediately frozen and stored at −80 °C until further analysis.

### 4.3. Determination of Antioxidant Enzyme Activities and Lipid Peroxidation

The enzymatic activities of SOD, CAT, total antioxidant capacity (T-AOC), acid phosphatase (ACP), and alkaline phosphatase (AKP) in the hepatopancreas of *P. clarkii* were quantified using a commercial assay kit (Beyotime, Shanghai, China) following the manufacturer’s protocols. SOD activity was determined using a WST-8 colorimetric method, with one unit defined as 50% inhibition of the reaction rate [78]. CAT activity was assessed by monitoring the decomposition rate of hydrogen peroxide into water and oxygen [11]. Both enzymes were expressed as U g^−1^ fresh weight (FW). T-AOC was measured via the ABTS radical scavenging assay and expressed as mmol g^−1^ FW [79]. ACP and AKP activities were determined using para-nitrophenyl phosphate (pNPP) as the substrate under optimal pH conditions, and the formation of para-nitrophenol was quantified spectrophotometrically at 405 nm [80,81]. Lipid peroxidation was evaluated by measuring the malondialdehyde (MDA) content, a degradation product of lipid peroxides that reacts with thiobarbituric acid (TBA) to form a red chromogenic adduct with a maximum absorption at 532 nm [82]. The MDA concentration was determined using a commercial kit (Nanjing Jiancheng Bioengineering Institute, Nanjing, China) and expressed as nmol g^−1^ FW.

### 4.4. Measurement of MT Content

The MT content in *P. clarkii* hepatopancreas tissues was quantified using a commercial enzyme-linked immunosorbent assay kit (Sinobestbio, Shanghai, China) as described previously [83,84,85]. The assay kit had a detection range of 0.1–800 nmol/L. Approximately 0.2 g of hepatopancreas tissues were homogenized in pre-chilled 0.9% saline and centrifuged at 3000× *g* for 10 min to obtain the supernatant. Microplate wells pre-coated with MT antigen were sequentially incubated with standards or samples, followed by incubation with horseradish peroxidase (HRP)-conjugated competitive antigen. After incubation and thorough washing to remove unbound components, 3,3′,5,5′-tetramethylbenzidine (TMB) substrate was added for color development. Absorbance was measured at 450 nm, and MT concentrations were determined using a standard calibration curve. Each treatment group included three biological replicates (*n* = 3).

### 4.5. Histopathological Examination

The hepatopancreas tissues of *P. clarkii* were rinsed three times in 1× PBS, cut into small pieces of approximately 1 cm, and fixed in 4% paraformaldehyde (Sangon Biotech, Shanghai, China) for 48 h, as described previously [86]. The fixed tissues were dehydrated using a graded ethanol series (75%, 85%, 90%, 95%, and 100%), treated with xylene, and embedded in paraffin. The paraffin-embedded tissues were sectioned into 5 μm thick slices, deparaffinized, rehydrated, stained with hematoxylin and eosin (H&E), and mounted with neutral balsam, as described previously [87]. All sections were examined for histopathological changes using an inverted microscope (Olympus IX73; Olympus Corporation, Tokyo, Japan).

### 4.6. Protein Extraction and Digestion

Hepatopancreas proteins from *P. clarkii* were extracted by homogenization in a protein lysis buffer containing 8 M urea and 1% SDS, supplemented with protease inhibitors as described previously [88,89]. After centrifugation at 12,000× *g* for 30 min at 4 °C, the supernatants were collected. Protein concentration was determined using a BCA assay kit (Thermo Scientific, Waltham, MA, USA). Subsequently, 100 μg of protein samples were reconstituted in 100 mM triethylammonium bicarbonate (TEAB) buffer. Reduction was performed using 10 mM tris(2-carboxyethyl)phosphine (TCEP) at 37 °C for 60 min, followed by alkylation in the dark using 40 mM iodoacetamide (IAM) at 25 °C for 40 min. Following centrifugation at 10,000× *g* for 20 min at 4 °C, the pellet was resuspended in 100 mM TEAB buffer and digested with trypsin (1:50 *w*/*w*, enzyme-to-protein) overnight at 37 °C. The resulting peptides were dried under vacuum, reconstituted in 0.1% trifluoroacetic acid (TFA), desalted using HLB cartridges, and concentrated using vacuum centrifugation. The peptide concentration was determined by measuring the absorbance at 280 nm using a NanoDrop One spectrophotometer (Thermo Fisher Scientific, Waltham, MA, USA).

### 4.7. Data-Independent Acquisition (DIA) Mass Spectrometry

Mass spectrometry analysis was performed using a Vanquish Neo UPLC system coupled to an Orbitrap Astral mass spectrometer (Thermo Fisher Scientific), as previously described [90,91]. Peptide separation was achieved on a uPAC High Throughput column (75 μm × 5.5 cm, Thermo Fisher Scientific). Mobile phase A consisted of 2% acetonitrile and 0.1% formic acid, and mobile phase B consisted of 80% acetonitrile and 0.1% formic acid. Peptides were separated using a gradient elution, and data acquisition was controlled using Xcalibur software (v.4.7; Thermo Fisher Scientific). DIA was performed using an Orbitrap Astral mass spectrometer, with full mass spectrometry scans acquired over an *m*/*z* range of 70–1050 and fragment ion spectra acquired over an *m*/*z* range of 150–2000 as previously described [90,92].

### 4.8. Protein Identification, Quantitation, and Functional Annotation

For protein identification, the raw data were processed using Spectronaut software (v.18.4 Biognosys AG, Schlieren, Switzerland) with reference to the *P. clarkii* genome [93]. For protein quantification, six peptides per protein and three fragment ions per peptide were selected as previously described [94,95]. Data analysis was performed using the following thresholds: false discovery rate (FDR) for proteins and peptides ≤ 0.01, peptide confidence ≥ 99%, and an extracted ion chromatogram (XIC) mass tolerance width ≤ 75 ppm. Shared and modified peptides were excluded from the analysis. DEPs were screened using the following criteria: a significance threshold of *p* < 0.05 (two-tailed Student’s *t*-test) and a fold change > 1.20 or <0.83 as described previously [96,97]. For functional annotation, DEPs were analyzed using Gene Ontology (GO) and Kyoto Encyclopedia of Genes and Genomes (KEGG) enrichment analyses, which were performed using TBtools (v.2.332) [98].

### 4.9. Quantitative RT-PCR (qRT-PCR) Analysis

Total RNA was extracted from hepatopancreas samples of the four experimental groups using a FastPure Complex Tissue/Cell Total RNA Isolation Kit (Vazyme, Nanjing, China), followed by DNase I treatment to eliminate residual genomic DNA. Purified RNA was reverse-transcribed into complementary DNA (cDNA) using a PrimeScript RT Reagent Kit with gDNA Eraser (TaKaRa, Dalian, China), following the manufacturer’s protocols. The qRT-PCR was performed on a CFX96 Real-Time PCR Detection System (Bio-Rad, Hercules, CA, USA) using TB Green Premix Ex Taq II (TaKaRa). The *18S ribosomal RNA* (*18S rRNA*) gene from *P. clarkii* served as an endogenous control [15,99]. Relative mRNA expression was calculated via the 2^−ΔΔCt^ method [100], with data derived from three independent biological replicates. The primers used for the qRT-PCR analysis are listed in Table S1.

### 4.10. Statistical Analysis

Data are expressed as mean ± standard deviation (SD) of three independent biological replicates. Graphical representations were generated using GraphPad Prism (v.10.0; GraphPad Software Inc., San Diego, CA, USA) and RStudio software (v.4.5.1). Statistical analyses were performed using SPSS software (v.27.0; SPSS Inc., Chicago, IL, USA). Prior to statistical analysis, the normality of the data distribution was assessed and confirmed. One-way analysis of variance (ANOVA) followed by Tukey’s multiple comparison test was used to assess the statistical differences among the groups. Statistical significance was set at *p* < 0.05, as previously described [15,97]. Statistical comparisons were performed within each time point, and temporal variations were interpreted based on observed trends.

## 5. Conclusions

This study provides integrated physiological and proteomic insights into the protective effects of exogenous MT against Cu-induced toxicity in *P. clarkii*. Cu exposure induced evident hepatopancreatic injury, as evidenced by pronounced oxidative stress and marked histopathological alterations. The administration of exogenous MT effectively alleviates these toxic effects by significantly enhancing antioxidant enzyme activities (SOD, CAT, and T-AOC), reducing MDA accumulation, and restoring Cu-suppressed endogenous MT levels and phosphatase activities (ACP and AKP). These physiological improvements may contribute to the preservation of hepatopancreatic structural integrity. Time-course analysis identified 24 h as the critical time point at which oxidative stress is fully activated and MT-mediated protective effects are most pronounced. Integrated proteomics reveals that MT may modulate multiple stress-related molecular pathways. MT may reverse Cu-induced dysregulation of pathways related to ABC transporters and membrane trafficking, thereby maintaining ion homeostasis and stabilizing mitochondrial biogenesis to sustain energy metabolism. Concurrently, MT suppresses the lysosomal degradation and apoptotic pathways, thereby preventing excessive catabolism and cell death. Notably, MT also enhances glutathione metabolism, strengthening the intrinsic detoxification capacity of *P. clarkii*. Collectively, these findings support a putative mechanistic framework for MT-mediated protection against Cu toxicity and provide a solid scientific foundation for its application as an effective bioprotective agent in aquaculture.

## Data Availability

The mass spectrometry proteomics data have been deposited to the ProteomeXchange Consortium via the iProX partner repository with the dataset identifier PXD075802.

## Supplementary Materials

The following supporting information can be downloaded at: https://www.mdpi.com/article/10.3390/ijms27125236/s1.
