# Supplementary material for: Physiological and Proteomic Insights into Melatonin-Mediated Regulation of Copper Toxicity in the Crayfish Procambarus clarkii
Source: Int J Mol Sci. 2026 Jun 9;27(12):5236. doi: 10.3390/ijms27125236 (PMC13299147; doi:10.3390/ijms27125236)
Supplement: Supplementary file 1 [file ijms-27-05236-s001.zip › Supplementary Figures.pdf]

Supplementary Figures

Figure S1

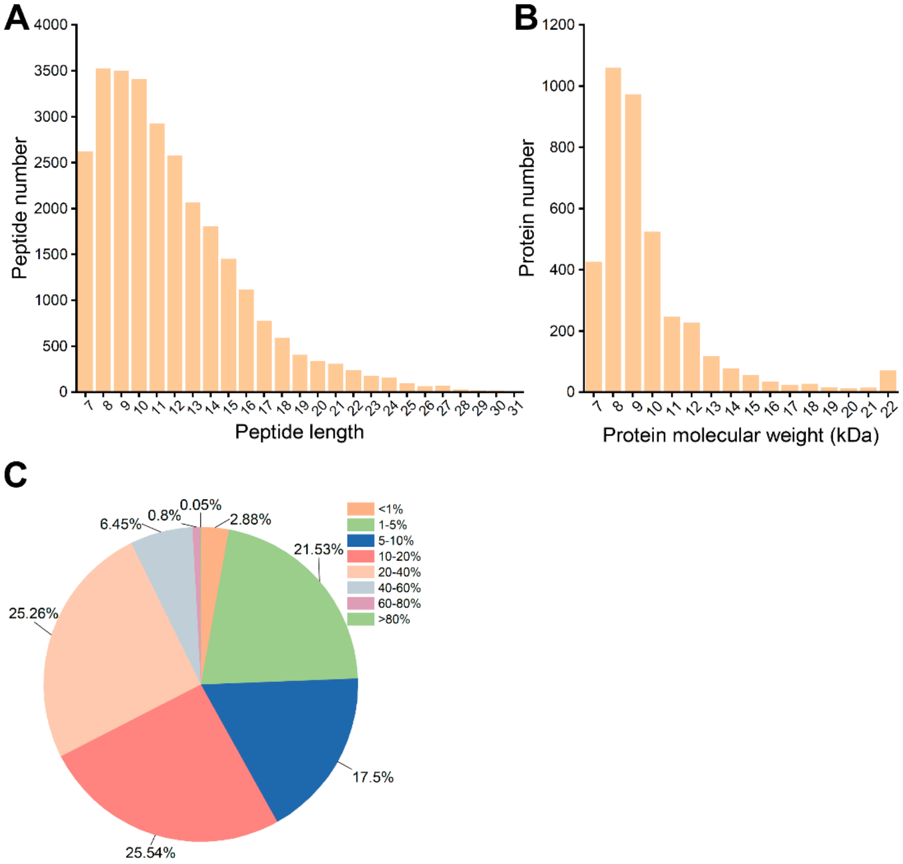

Figure S1. Proteomic profiling of the hepatopancreas in *Procambarus clarkii* following 24 h exposure to copper (Cu) and melatonin (MT). (A) Length distribution of identified peptide fragments. (B) Molecular weight distribution of detected proteins. (C) Distribution of protein sequence coverage across all identified proteins.

Figure S2

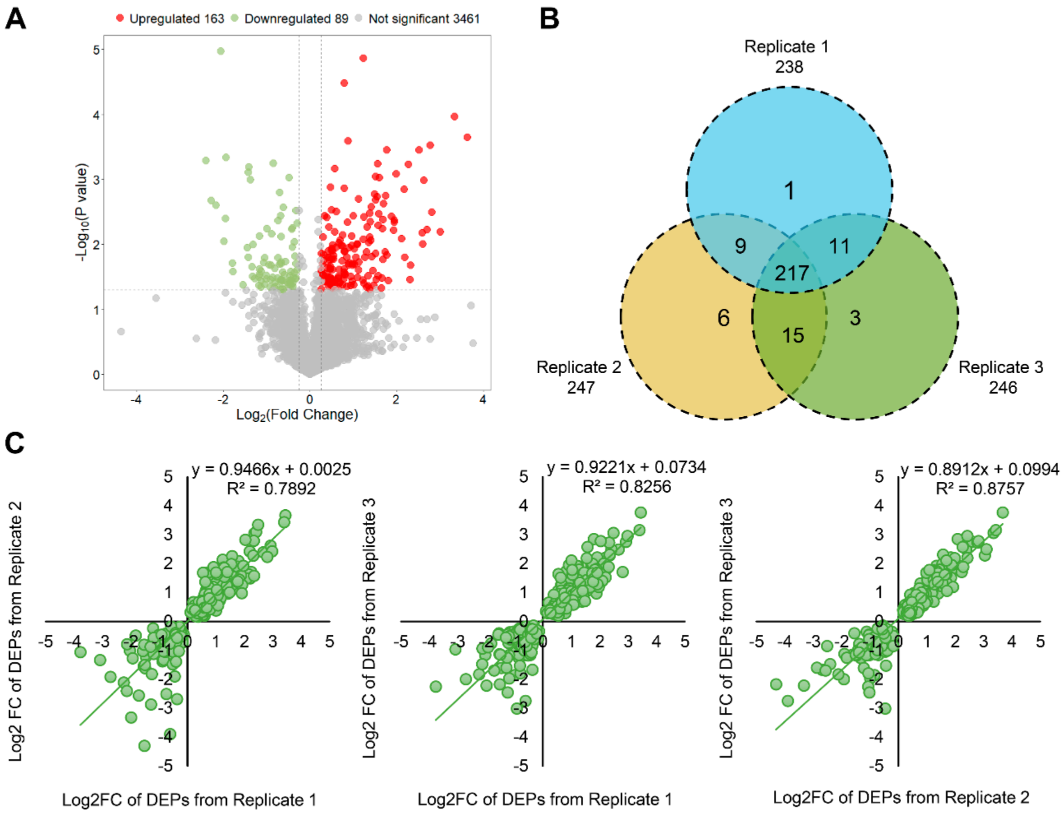

Figure S2. Identification and reproducibility assessment of differentially expressed proteins (DEPs) between Cu-exposed and control *P. clarkii*. (A) Volcano plot depicting DEPs between the Cu-treated and control groups, based on $\log_2$-transformed fold-change (FC) values and corresponding $-\text{Log}_{10}$ P-values. Proteins exhibiting significant upregulation and downregulation are highlighted in red and green, respectively, whereas nonsignificant proteins are shown in gray. (B) Venn diagram illustrating the overlap of DEPs among three independent biological replicates following 24 h of Cu exposure, with 217 DEPs consistently identified across all replicates. (C) Pairwise correlation analysis of Log_2FC values among the three biological replicates reveals strong reproducibility and high concordance of the proteomic data.

Figure S3

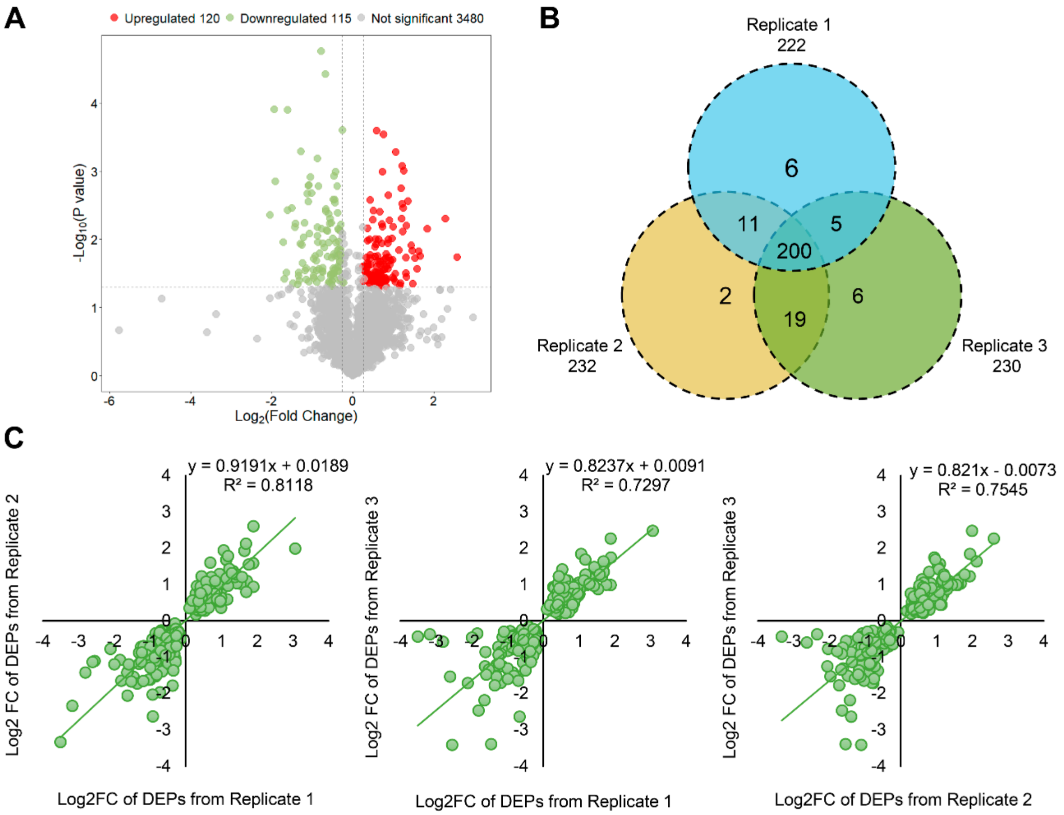

Figure S3. Identification and reproducibility assessment of differentially expressed proteins (DEPs) between Cu+MT-treated and control *P. clarkii*. (A) Volcano plot depicting DEPs between the Cu+MT-treated and control groups, based on $\log_2$-transformed fold-change (FC) values and corresponding $-\text{Log}_{10}$ P-values. Proteins significantly upregulated and downregulated are highlighted in red and green, respectively, while nonsignificant proteins are shown in gray. (B) Venn diagram illustrating the overlap of DEPs among three independent biological replicates following 24 h of Cu+MT exposure, with 200 DEPs consistently identified across all replicates. (C) Pairwise correlation analysis of Log_2FC values among the three biological replicates reveals high reproducibility and strong concordance of the proteomic dataset.

Figure S4

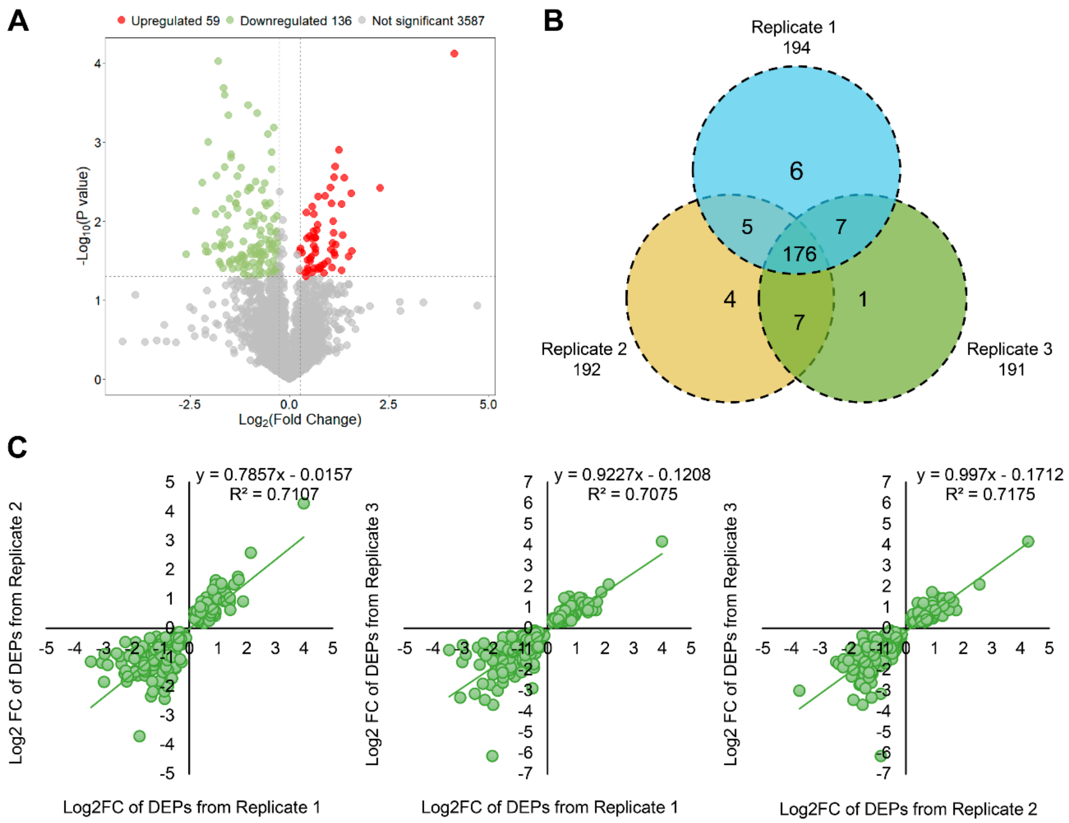

Figure S4. Identification and reproducibility assessment of differentially expressed proteins (DEPs) between Cu+MT-treated and Cu-treated *P. clarkii*. (A) Volcano plot depicting DEPs between the Cu+MT-treated and Cu-treated groups, based on log_2-transformed fold-change (FC) values and corresponding $-Log_{10}$ P-values. Proteins significantly upregulated and downregulated are highlighted in red and green, respectively, while nonsignificant proteins are shown in gray. (B) Venn diagram illustrating the overlap of DEPs among three independent biological replicates after 24 h of Cu exposure, with 176 DEPs consistently identified across all replicates. (C) Pairwise correlation analysis of Log_2FC values among the three biological replicates reveals high reproducibility and strong concordance of the proteomic dataset.

Figure S5

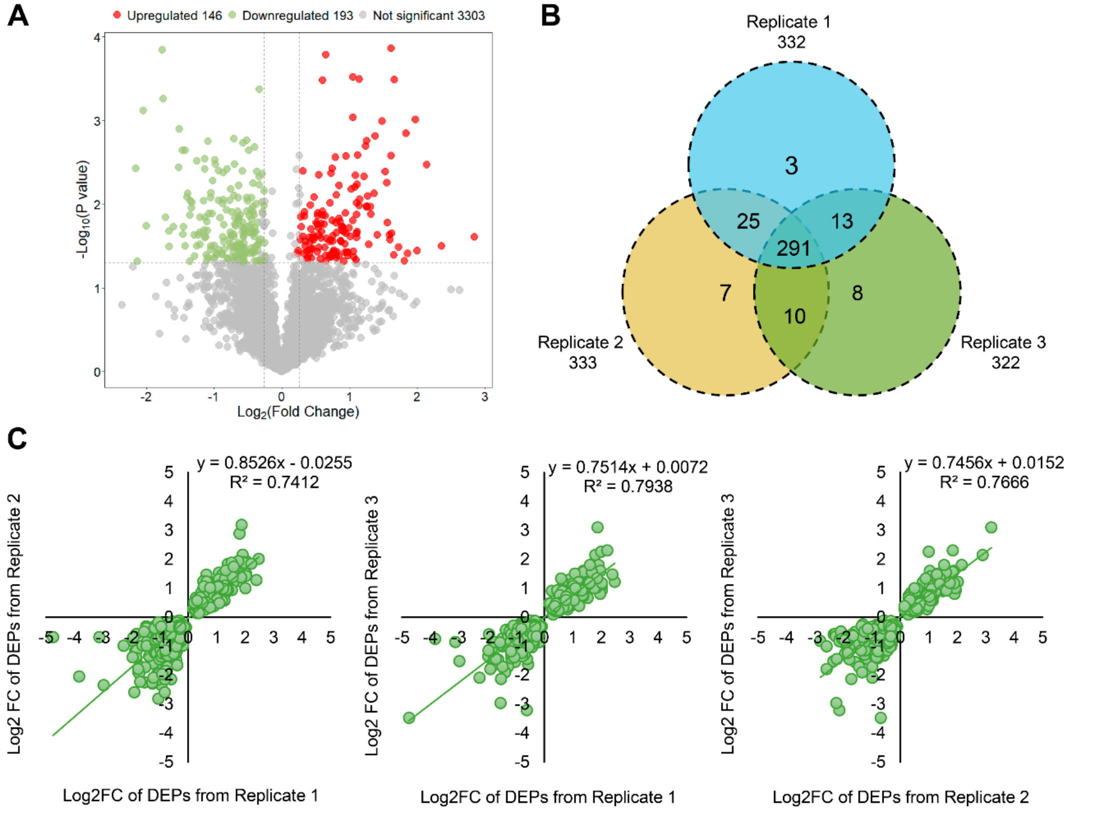

Figure S5. Identification and reproducibility assessment of differentially expressed proteins (DEPs) between MT-treated and control *P. clarkii*. (A) Volcano plot depicting DEPs between the MT-treated and control groups, based on $\log_2$-transformed fold-change (FC) values and corresponding $–\mathrm{Log}_{10}$ P-values. Proteins significantly upregulated and downregulated are highlighted in red and green, respectively, while nonsignificant proteins are shown in gray. (B) Venn diagram illustrating the overlap of DEPs among three independent biological replicates after 24 h of MT exposure, with 291 DEPs consistently identified across all replicates. (C) Pairwise correlation analysis of Log_2FC values among the three biological replicates reveals high reproducibility and strong concordance of the proteomic dataset.
